# FSH regulates glucose‐stimulated insulin secretion: A bell‐shaped curve effect

**DOI:** 10.1111/1753-0407.13546

**Published:** 2024-04-10

**Authors:** Hong Zhu, Guolian Ding, Hefeng Huang

**Affiliations:** ^1^ Obstetrics and Gynecology Hospital Institute of Reproduction and Development, Fudan University Shanghai China; ^2^ Research Units of Embryo Original Diseases Chinese Academy of Medical Sciences Shanghai China; ^3^ Shanghai Key Laboratory of Reprodction and Development Fudan University Shanghai China; ^4^ Key Laboratory of Reproductive Genetics (Ministry of Education) Zhejiang University School of Medicine Hangzhou China

Follicle‐stimulating hormone (FSH), a classical hormone derived from the pituitary, primarily affects the gonads and regulates the reproductive process.^1^ FSH consists of an α and a β subunit, with the β subunit specifically binding to its G protein‐coupled receptor (GPCR), FSHR.^2^ The FSHR mediates the transduction of the FSH‐induced signal. According to the recent work published in *Nature Communications*, FSH, through its receptor, regulates glucose‐stimulated insulin secretion (GSIS) in pancreatic islets, and high levels of FSH play important roles in postmenopausal diabetes in females.^3^

An increasing body of evidence has demonstrated that FSH and its receptor FSHR also have extragonadal effects, including the regulation of fat accumulation, bone mass, and cognitive function.^4^, ^5^, ^6^, ^7^ However, limited research has been focused on the effect of FSH on metabolism. The pancreas is an important endocrine organ in regulating glucose metabolism. First, the authors explored whether FSHR was expressed in pancreas. They identified the expression of FSHR in human pancreas, mouse pancreatic islets, and the mouse insulinoma cell line MIN6. The expression of FSHR in pancreatic islets strongly suggests an association between FSHR and the endocrine function. In order to explore the function of FSH and FSHR on pancreatic islets, the authors established a conventional Fshr^−/−^(knockout [KO]) mouse model. Blocking FSH signaling through Fshr KO resulted in impaired glucose tolerance. In this model, Fshr KO led to an increase in serum FSH levels as well as a decrease in serum estrogen levels. Females with Fshr KO administrated with estrogen also displayed impaired glucose tolerance. Furthermore, the authors generated a mouse model with specific deletion of Fshr in the pancreas (Fshr CKO), which showed no significant alterations in serum FSH and estrogen levels. Similarly, female Fshr CKO mice exhibited impaired glucose tolerance. The phenotype of glucose intolerance was also observed in male mice with Fshr KO and CKO male mice.

Glucose intolerance is primarily caused by impaired insulin secretion and action. The authors evaluated peripheral insulin action and found there was no significant insulin resistance in Fshr KO and CKO mice. However, decreased insulin secretion was observed in Fshr KO and CKO mice. In vitro, treatment of mouse pancreatic islets and MIN6 cells with FSH did not result in any significant changes in Ins1 and Ins2 mRNA levels or insulin content, suggesting that the effect of FSH on glucose tolerance was due to insulin secretion, not insulin synthesis. Interestingly, the authors discovered that FSH alone, in the absence of glucose, did not stimulate insulin secretion. FSH regulated GSIS in a bell curve manner. FSH promoted GSIS as FSH levels increased within the range of <10 IU/L. However, the promoting effect on GSIS was inhibited as FSH levels increased beyond 10 IU/L.

Typically, the FSHR has been shown to directly activate G proteins, thereby intensifying the FSH signal action.^8^ Previous studies have shown that G proteins mediate the activation of various signaling pathways, including Gαs/cyclic adenosine monophosphate (cAMP) and intracellular Ca^2+^‐related signaling.^9^ Interestingly, cAMP and intracellular Ca^2+^ signals play a crucial role in the exocytosis of insulin granules.^10^, ^11^ In vitro, FSH at concentrations <10 IU/L significantly increased the intracellular cAMP levels, protein kinase A (PKA) activity, and intracellular Ca^2+^ level in a concentration‐dependent manner. However, high concentrations of FSH (10–100 IU/L) decreased the intracellular cAMP levels, PKA activity, and intracellular Ca^2+^ levels in a dose‐dependent way.

FSHR regulates intracellular cAMP levels by coupling with Gαs or Gαi protein in gonadal cells.^8^, ^11^ In the absence of FSH, Gαs and Gαi inhibitors did not affect insulin secretion and intracellular cAMP levels. At low FSH (10 IU/L) or high FSH (100 IU/L), the Gαs inhibitor led to a significant decrease in insulin secretion and intracellular cAMP content. However, Gαi inhibitor increases insulin secretion and intracellular cAMP levels only under 16.7 mM glucose with 100 IU/L FSH. Originally, it was thought that each GPCR signals through a single cognate G protein class to initiate the “canonical” signaling of the receptor. However, some studies have also shown that receptors can couple to more than one Gα protein to initiate noncanonical GPCR signaling.^12^ The authors showed that FSHR might simultaneously couple with Gαs and Gαi proteins, depending on FSH levels.

This noncanonical FSHR signaling pattern may help explain the bell curve effect of FSH on GSIS. Previous studies have reported that high FSH levels in postmenopausal women are associated with bone loss, visceral adiposity, and cognitive impairment. In this study, the authors uncovered a critical extragonadal role of FSH in the regulation of GSIS in pancreatic islets. The bell curve effect suggests that FSH has dual effects. Before perimenopause, low levels of FSH promote an increase in GSIS. However, this promoting effect is inhibited during high FSH levels. Furthermore, the authors established an ovariectomized (OVX) mouse model to mimic the hormonal status of postmenopausal women. In OVX mice, both FSH and luteinizing hormone (LH) increased and estrogen decreased. The OVX mice were administered the gonadotropin‐releasing hormone agonist (GnRHa) to reduce FSH and LH levels. Subsequently, OVX + GnRHa mice were injected with exogenous FSH to mimic postmenopausal high serum FSH levels and given estrogen to maintain relatively normal serum estrogen levels. In the OVX model, the authors demonstrated that high levels of FSH alone result in impaired glucose tolerance and insulin secretion.

In summary, this study not only reports the expression of FSHR on pancreatic islets but also characterizes the novel role of FSH as a dual regulator of GSIS. FSH regulates GSIS through the FSHR‐Gαs/Gαi‐intracellular cAMP and Ca^2+^ signaling pathway (Figure 1). Future research is necessary to identify the molecular targets that can block FSH signaling, which will provide new avenues for therapeutic strategies of postmenopausal diabetes.

**FIGURE 1:**
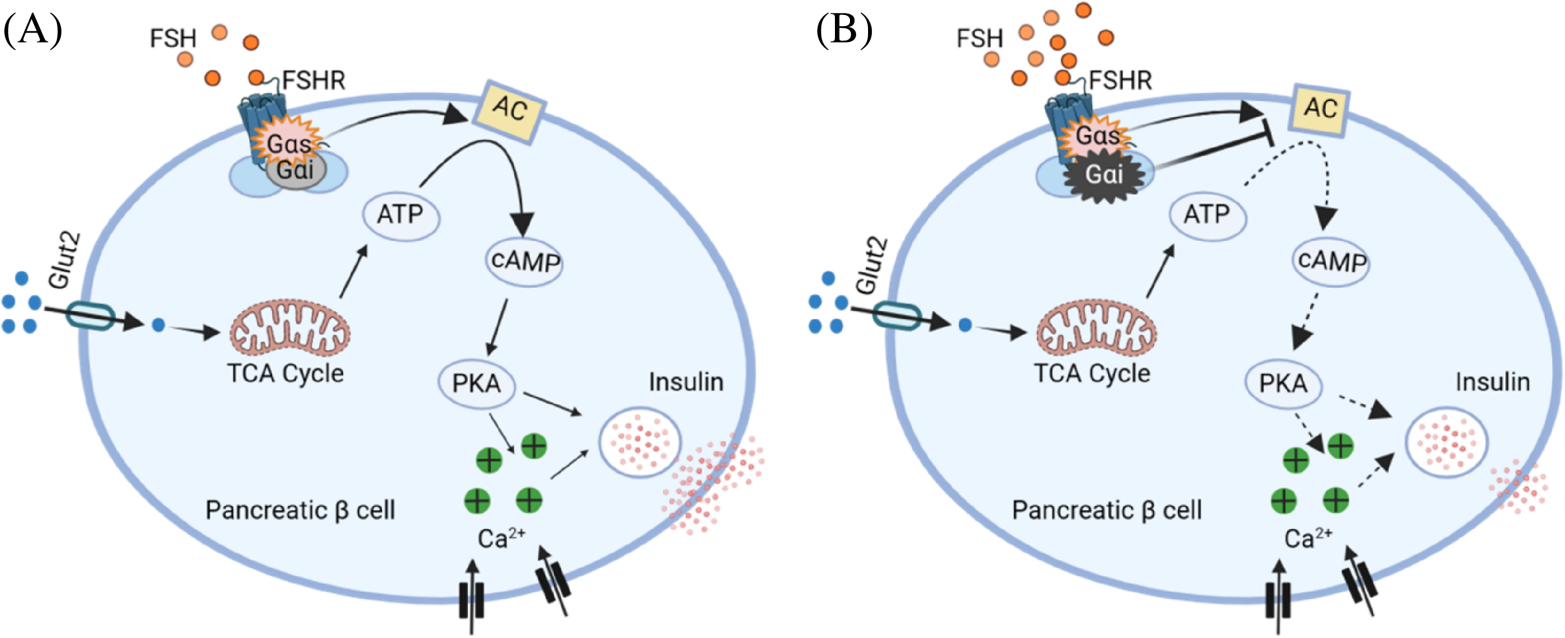
Follicle‐stimulating hormone (FSH) regulates glucose‐stimulated insulin secretion (GSIS) through FSHR. (A) Low levels of FSH (<10 IU/L), through FSHR, activate Gαs protein, causing adenylatecyclase (AC) activation, promoting the cAMP/PKA pathway and intracellular Ca^2+^ levels to enhance GSIS. (B) High levels of FSH (>10 IU/L), through FSHR, activate Gαi protein, simultaneously, inhibiting the activation of AC, attenuating the cyclic adenosine monophosphate/protein kinase A (cAMP/PKA) pathway and intracellular Ca^2+^ signaling, inhibiting the promoting effect of GSIS. ATP, adenosine triphosphate; FSHR, follicle‐stimulating hormone receptor; Glut2, glucose transporter 2; TCA cycle, tricarboxylic acid cycle.

## CONFLICT OF INTEREST STATEMENT

The authors declare that they have no conflict of interest.
